# Structural and Oxidation State Alternatives in Platinum and Palladium Complexes of a Redox‐Active Amidinato Ligand

**DOI:** 10.1002/chem.202003636

**Published:** 2021-01-12

**Authors:** Fabian Ehret, Vasileios Filippou, Svenja Blickle, Martina Bubrin, Stanislav Záliš, Wolfgang Kaim

**Affiliations:** ^1^ Institut für Anorganische Chemie Universität Stuttgart Pfaffenwaldring 55 70550 Stuttgart Germany; ^2^ J. Heyrovský Institute of Physical Chemistry, v.v.i. Academy of Sciences of the Czech Republic Dolejškova 3 18223 Prague Czech Republic

**Keywords:** electron transfer, oxidation states, radical ligands, spectroelectrochemistry, spin distribution

## Abstract

Reaction of [Pt(DMSO)_2_Cl_2_] or [Pd(MeCN)_2_Cl_2_] with the electron‐rich LH=N,N’‐bis(4‐dimethylaminophenyl)ethanimidamide yielded mononuclear [PtL_2_] (**1**) but dinuclear [Pd_2_L_4_] (**2**), a paddle‐wheel complex. The neutral compounds were characterized through experiments (crystal structures, electrochemistry, UV‐vis‐NIR spectroscopy, magnetic resonance) and TD‐DFT calculations as metal(II) species with noninnocent ligands L^−^. The reversibly accessible cations [PtL_2_]^+^ and [Pd_2_L_4_]^+^ were also studied, the latter as [Pd_2_L_4_][B{3,5‐(CF_3_)_2_C_6_H_3_}_4_] single crystals. Experimental and computational investigations were directed at the elucidation of the electronic structures, establishing the correct oxidation states within the alternatives [Pt^II^(L^−^)_2_] or [Pt^.^(L )_2_], [Pt^II^(L^0.5−^)_2_]^+^ or [Pt^III^(L^−^)_2_]^+^, [(Pd^II^)_2_(μ‐L^−^)_4_] or [(Pd^1.5^)_2_(μ‐L^0.75−^)_4_], and [(Pd^2.5^)_2_(μ‐L^−^)_4_]^+^ or [(Pd^II^)_2_(μ‐L^0.75−^)_4_]^+^. In each case, the first alternative was shown to be most appropriate. Remarkable results include the preference of platinum for mononuclear planar [PtL_2_] with an *N*‐Pt‐N bite angle of 62.8(2)° in contrast to [Pd_2_L_4_], and the dimetal (Pd_2_^4+^→Pd_2_^5+^) instead of ligand (L^−^→L ) oxidation of the dinuclear palladium compound.

## Graphical abstract

**Qualitatively different** structures and electron transfer behavior have been established for platinum and palladium complexes [PtL_2_]^0/+^ and [Pd_2_L_4_]^0/+^ with a redox‐active amidinato ligand L^−^ */*L^.^.

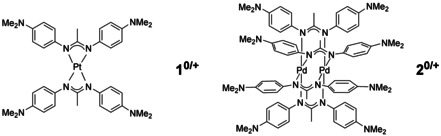

## Introduction

**Scheme 1:**
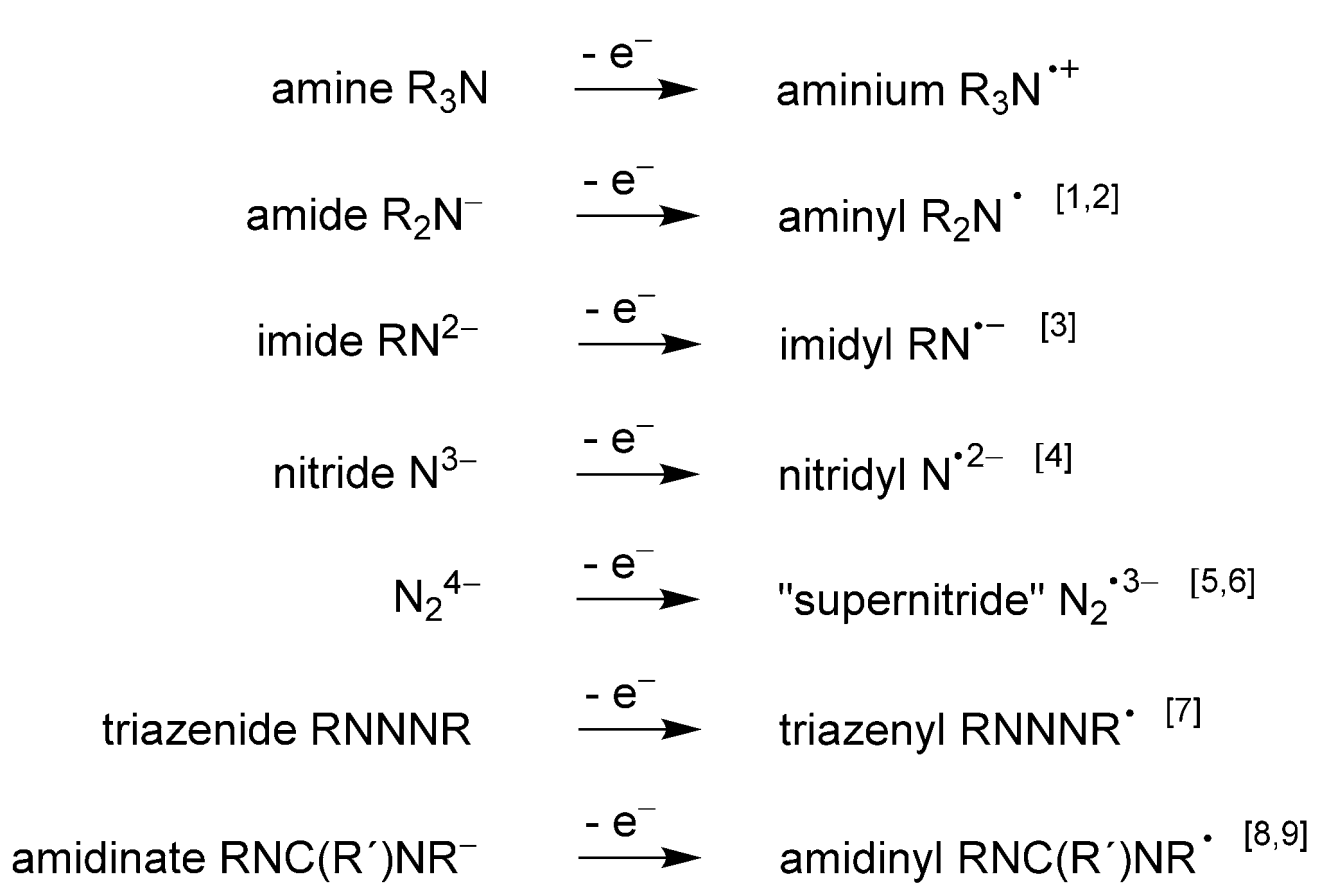
One‐electron oxidation of representative nitrogen ligands.

Open‐shell nitrogen ligands can occur in rather different forms, as illustrated by the non‐exhaustive list of Scheme 1.

**Scheme 2:**
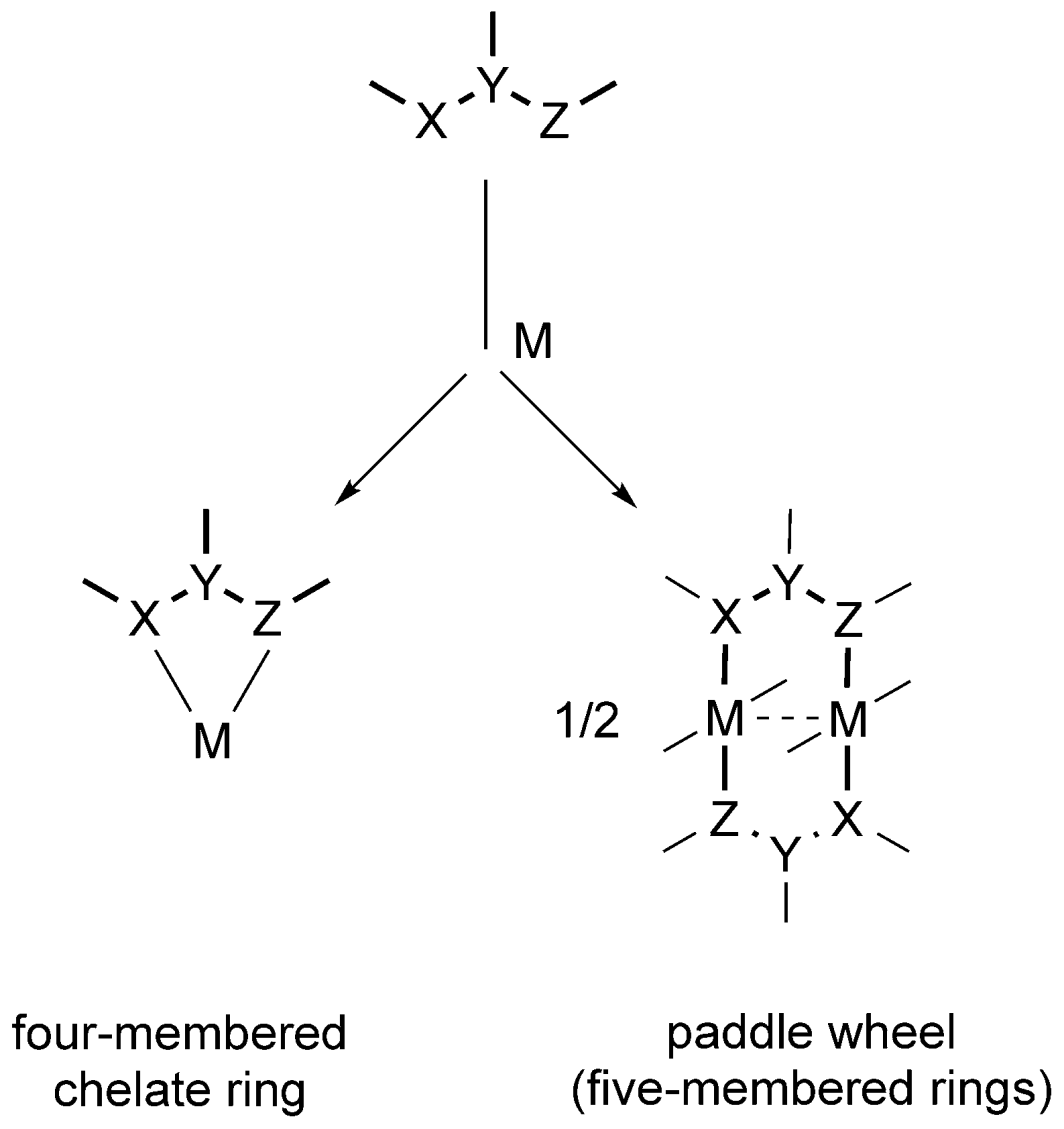
Coordination alternatives relevant to this article.

These fairly recently recognized species with potentially noninnocent behavior can play important roles in chemical reactions, including catalytic processes, in materials with special physical properties, and for mimics of biochemical transformations.^[10]^ A new *N*‐coordinating type of radical ligand has been presented by us recently[^8^, ^9^] in the form of amidinyl radicals [RNC(R’)NR]^.^=L , *R*=4‐dimethylaminophenyl, R’=CH_3_.^[8]^ With ruthenium complex fragments there exists an ambiguity in oxidation state formulation, Ru^III^(L^−^) vs. Ru^II^(L ), for certain oxidized forms, depending on the co‐ligands.^[9]^ Whereas ruthenium(III)‐tolerating 2,2’‐bipyridine co‐ligands favor the more conventional formulation Ru^III^(L^−^) with amidinato and EPR‐detectable ruthenium(III) components, the use of areneruthenium species was shown by EPR to stabilize ruthenium(II) and thus leave only the L side available for oxidation, producing Ru^II^(L ) complexes of amidinyl radical ligands.^[9]^ This ambiguity which was similarly shown for triazenido/triazenyl systems^[7]^ and was also discussed with respect to the NO_2_^−^/NO_2_^.^ pair[^7^, ^11^] led us to explore the platinum and palladium coordination chemistry of the deprotonated form of the electron‐rich amidine N,N’‐bis(4‐dimethylaminophenyl)ethanimidamide=HL, probing the formation of potentially noninnocent^[10]^ amidinato (L^−^) or amidinyl (L ) ligands. Platinum and palladium compounds are distinguished inter alia by stable planar M^II^(d^8^) configurations, a reluctance to adopt odd‐electron configurations (M^I^ or M^III^), and by their capacity to form four‐membered chelate rings^[12]^ or quadruply bridged metal‐metal bonded entities with paddle‐wheel arrangement[^13^, ^14^, ^15^] (Scheme 2), both kinds favored by three‐center bidentate ligands.

## Results and Discussion

### Synthesis and Structures

**Scheme 3:**
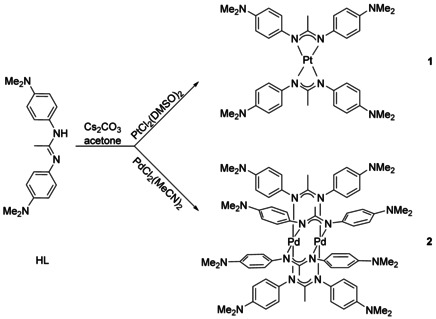
Reactions yielding different kinds of products.

The reaction of HL in acetone/Cs_2_CO_3_ with either *cis*‐[Pt(DMSO)_2_Cl_2_] or *cis*‐[Pd(MeCN)_2_Cl_2_] yields two different kinds of products, mononuclear [PtL_2_], **1**, and dinuclear [Pd_2_L_4_], **2** (Scheme 3).

Variation of the reaction conditions such as temperature, reaction time and solvents did not change the divergent product distribution. The dipalladium complex could be isolated in cationic crystalline form as [Pd_2_L_4_][B{3,5‐(CF_3_)_2_C_6_H_3_}_4_] via oxidation with the ferrocenium compound of tetrakis(3,5‐bis(trifluoromethyl)phenyl)borate, a similar procedure for **1** did not yield a crystalline species.

**Figure 1:**
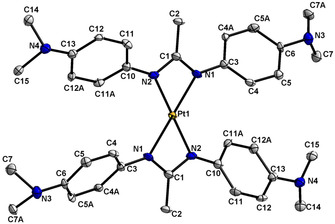
Molecular structure of [PtL_2_] in the crystal of [PtL_2_]×CH_2_Cl_2_. Hydrogen atoms are omitted for clarity, displacement ellipsoids drawn at 50 % probability. Bond lengths (Å) and angles (°): Pt1−N2 2.049(5), Pt1−N1 2.056(6), Pt−C1 2.527(7); N2‐Pt1‐N1 117.2(2), N2‐Pt1‐N1#1 62.8(2), N1#1‐Pt1‐N1 180.0(5).

**Figure 2:**
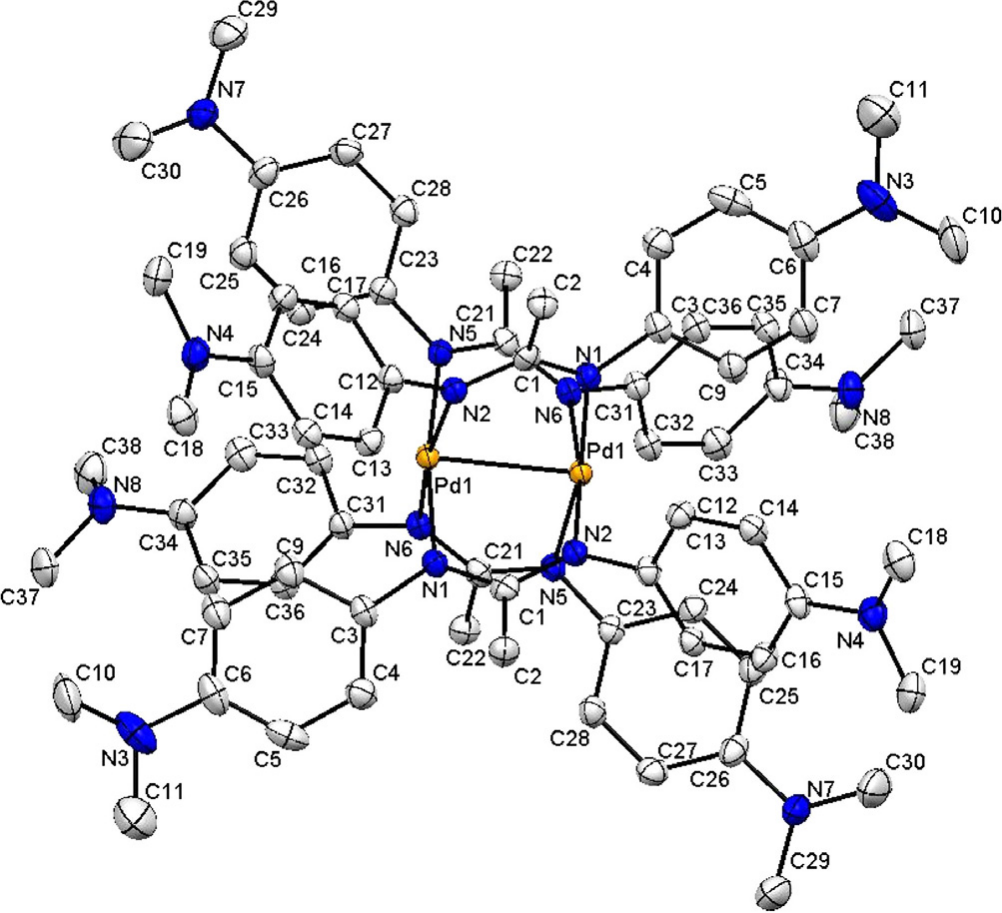
Molecular structure of [Pd_2_L_4_] in the crystal (molecule with „Pd1“, for crystallographically independent molecule with “Pd2” see Figure S1). Hydrogen atoms are omitted for clarity, displacement ellipsoids drawn at 50 % probability. Bond lengths (Å) and angles (°): Pd1−Pd1#1 2.5432(4), Pd1−N1 2.049(5), Pd1−N2 2.047(2), Pd1−N5 2.053(2), Pd1−N6 2.049(2); N2‐Pd1‐N6 171.83(9), N1‐Pd1‐N5 170.81(9); *N*‐Pd1‐N 88–92, *N*‐Pd1‐Pd1#1 85–87.

**Figure 3:**
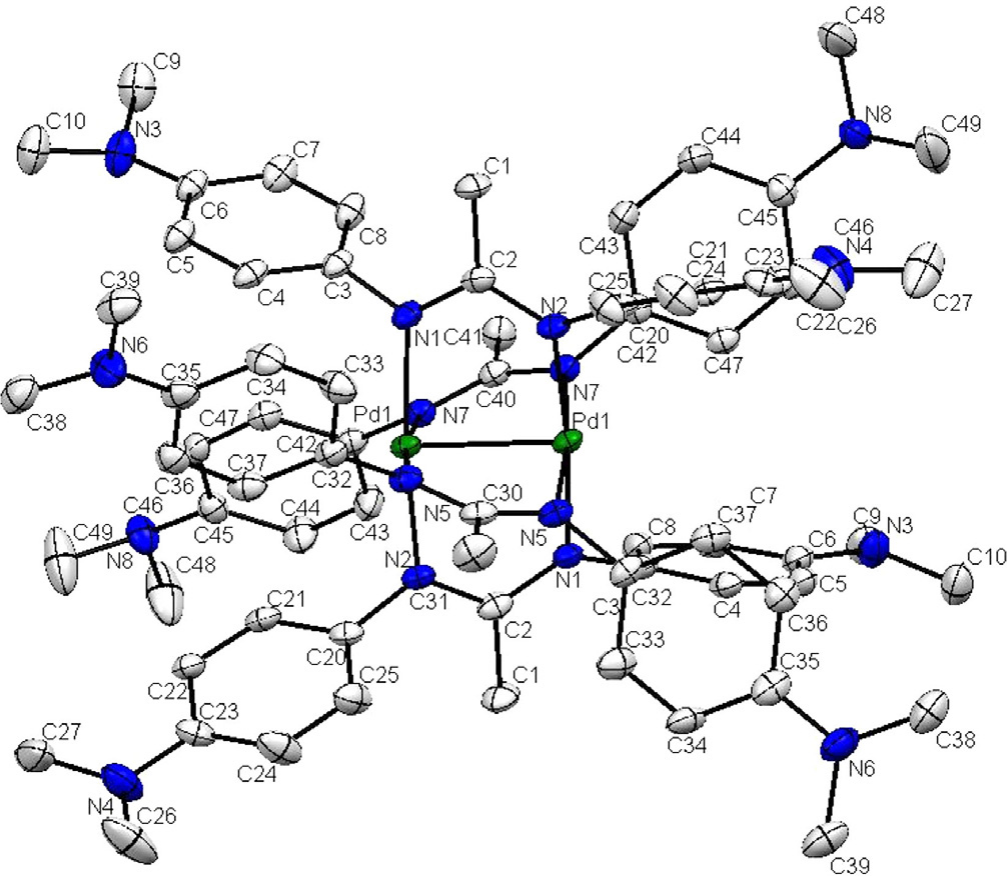
Molecular structure of [Pd_2_L_4_]^+^ in the crystal of of [Pd_2_L_4_][B{3,5‐(CF_3_)_2_C_6_H_3_}_4_]. Hydrogen atoms are omitted for clarity, displacement ellipsoids drawn at 50 % probability. Bond lengths (Å) and angles (°): Pd1−Pd1#1 2.4384(9), Pd1−N1 2.033(2), Pd1−N2 2.035(2), Pd1−N5 2.028(2), Pd1−N7 2.042(2); N1‐Pd1‐N2 173.84(10), N5‐Pd1‐N7 173.61(10); N‐Pd1‐N 89–91, N‐Pd1‐Pd1#1 86–87.2.

The products **1**, **2** and **2^+^** were characterized spectroscopically (see Supporting Information), electrochemically and via X‐ray diffraction (Tables S1–S4). The molecular structures of **1**, **2** and **2^+^** are shown in Figures 1–3 and S1, and some essential structural parameters are listed in Tables S2–S4 together with DFT‐calculated values for geometry‐optimized species.

The platinum(II) complex **1** exhibits the characteristic planar configuration at a *d* ^*8*^ configurated metal. While the bond lengths are not unusual, the four‐membered chelate rings include rather small N‐Pt‐N bite angles of about 63°, leaving large values of 117° and 180° for the other two N‐Pt‐N angles (Table S2). Despite this unusual bonding situation the experimental structure shows a planar arrangement of the platinum and attached N atoms which points to a d^[8]^ Pt^II^ configuration. The DFT calculation results reproduce the experimental geometry (Table S2). Therefore the system is best described as [Pt^II^(L^−^)_2_] and not as [Pt^0^(L )_2_] which might have been anticipated considering the facile oxidation of L^−^.^[8]^

With the palladium precursor Pd(MeCN)_2_Cl_2_ the reaction (Scheme 3) leads to quite a different product, the dinuclear paddle‐wheel complex [Pd_2_L_4_] with a Pd‐Pd distance of about 2.55 Å (Tables S3,S4). Related dipalladium compounds with similar metal‐metal distances, planar Pd configuration and a Pd_2_^4+^ description have been reported,[^13^, ^14^] suggesting an oxidation state formulation [Pd^II^(L^−^)_4_] for **2**, as confirmed by spectroscopy and DFT calculations (vide infra). The paddle‐wheel ligands at the PdPd axis are slightly twisted (e.g. 17.5° for N1‐Pd‐Pd‐N4 (15.7° from DFT calculations)).

The cation [Pd_2_L_4_]^+^ obtained after oxidation with ferrocenium exhibits a shortened metal‐metal bond of about 2.44 Å, as has been similarly noted for Pd_2_^5+^ cases,[^13^, ^15^] which is attributed to the removal of an antibonding electron.^[13]^ The Pd−N bonds are also shortened (Table S3). Both the neutral and the cationic dipalladium complex can be reproduced in their structures by DFT calculations (Table S3), lending support to the DFT‐based assignments of electronic absorption and EPR data discussed in the following.

### Cyclic voltammetry, EPR and UV‐vis‐NIR spectroelectrochemistry

**Figure 4:**
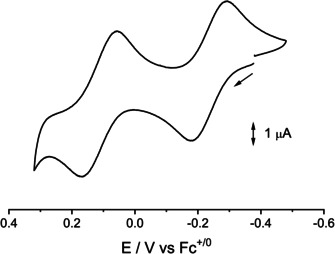
Cyclic voltammogram of **1** in CH_2_Cl_2_/0.1 m Bu_4_NPF_6_ (scan rate 100 mV s^−1^).

**Figure 5:**
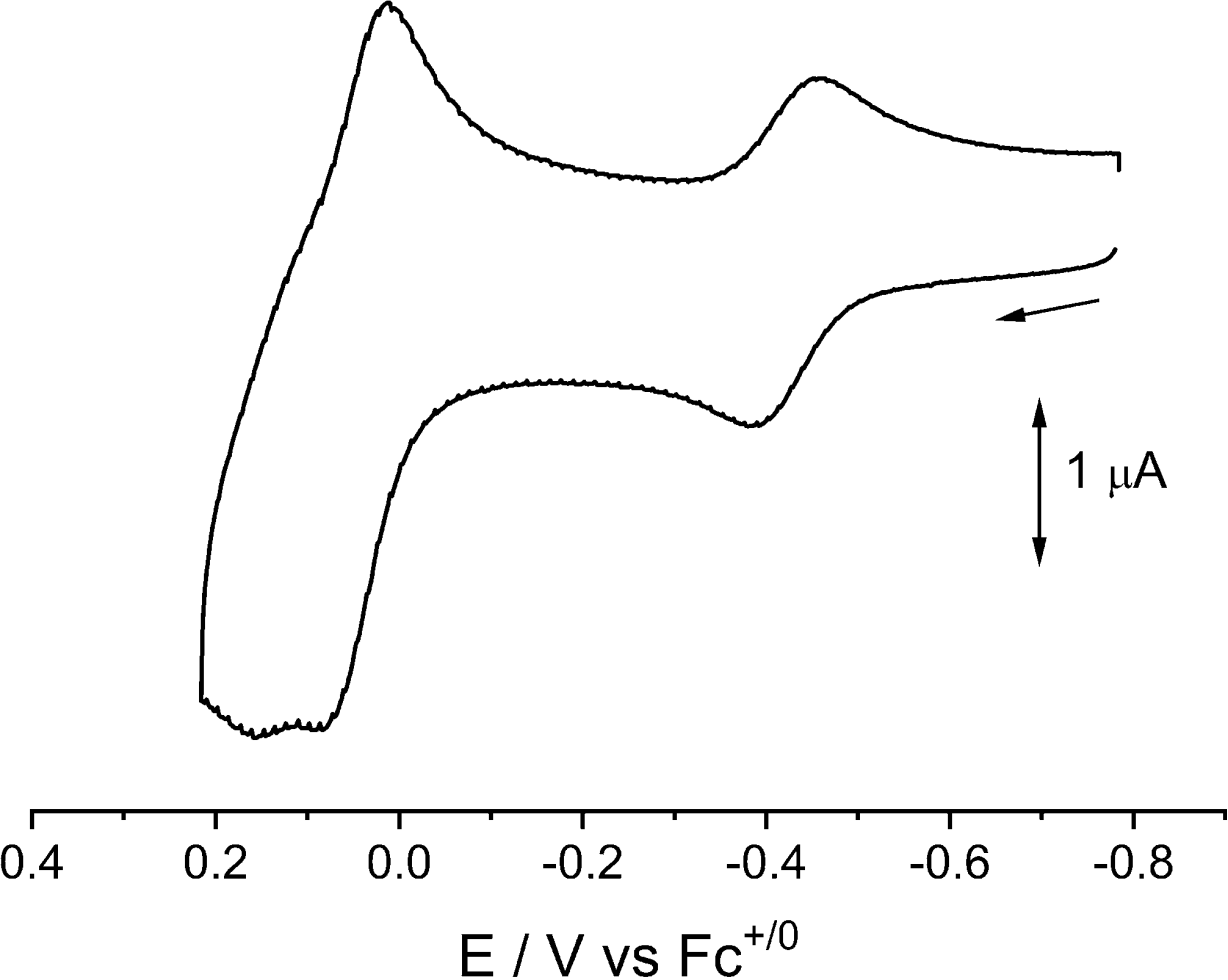
Cyclic voltammogram of **2** in CH_2_Cl_2_/0.1 m Bu_4_NPF_6_ (scan rate 100 mV s^−1^).

Compounds **1** and **2** each undergo one reversible first oxidation (1e) and a second oxidation involving one (**1^+^/1^2+^**) or more electrons **(2^+^/2 n^+^**) in CH_2_Cl_2_/0.1 m Bu_4_NPF_6_ (Figures 4,5). The corresponding E(1/2) values (in V vs. Fc^+/o^) are −0.23 and 0.13 V for **1 n^+^** and −0.42 and 0.02 V for **2 n^+^**. In agreement with the following experimental and computational results, compound **1** is assumed to undergo two stepwise one‐electron oxidation processes, attributed to the coplanar ligands (L^−^→L ), whereas the dipalladium compound **2** shows a one‐electron oxidation (Pd_2_^4+^→Pd_2_^5+^), followed by a larger wave system, tentatively attributed to multiple ligand oxidation.

**Figure 6:**
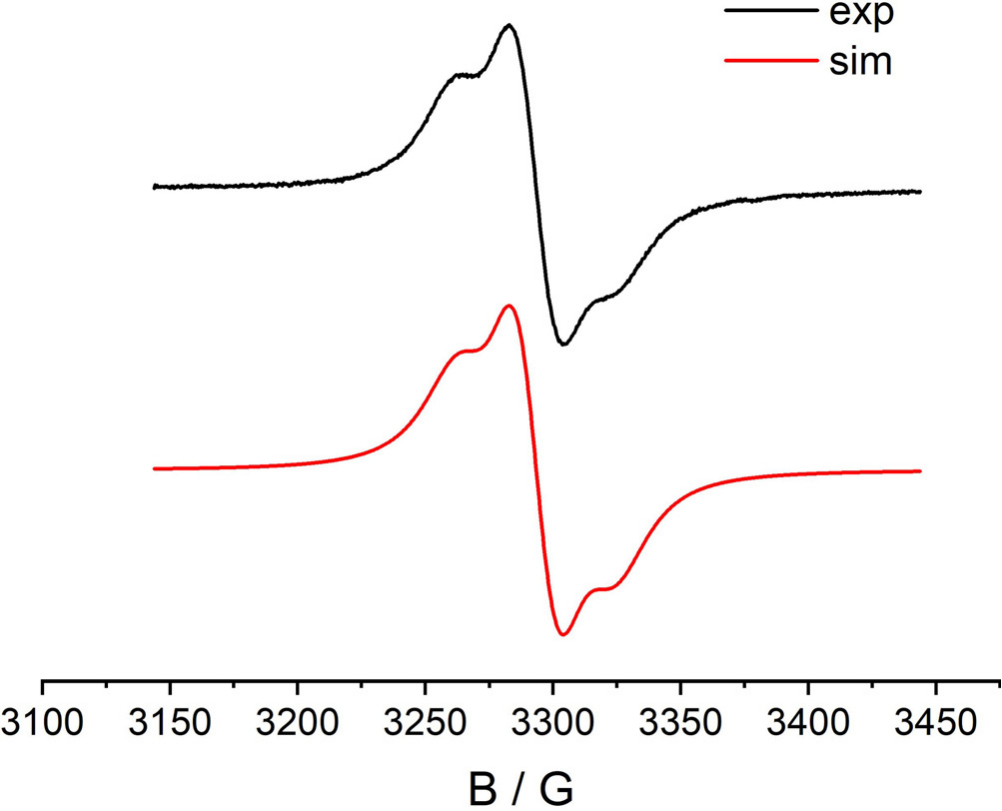
EPR spectrum of anodically generated **1^+^** in CH_2_Cl_2_/0.1 m Bu_4_NPF_6_ at 298 K.

**Figure 7:**
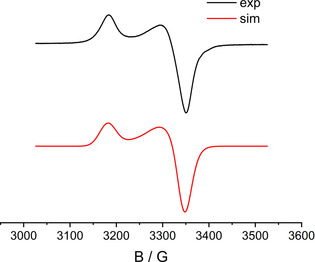
EPR spectrum of **1^+^** in CH_2_Cl_2_/0.1 m Bu_4_NPF_6_ at 110 K.

**Figure 8:**
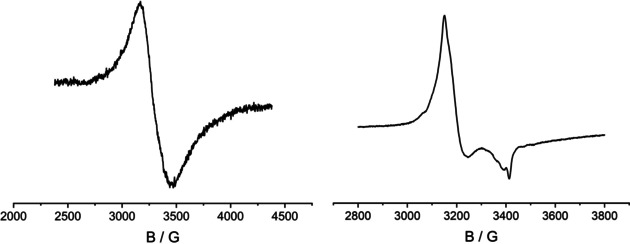
EPR spectrum of anodically generated **2^+^** in CH_2_Cl_2_/0.1 m Bu_4_NPF_6_ at 298 K (left) and of **2^+^** in CH_2_Cl_2_ at 110 K from oxidation with Fc(PF_6_) (right).

The comproportionation constants of about *K_c_*≈10^7^ for the cations **1^+^** and **2^+^** and the reversibility of the first oxidation processes allowed us to study the EPR properties of the paramagnetic intermediates (Figures 6–8, Table 1).

**Table 1:** EPR data for **1^+^** and **2^+^** in CH_2_Cl_2_/0.1 m Bu_4_NPF_6_.

Complex	*g_iso_* (298 K)	*g_1_*, *g_2_*, *g_3_* (110 K)	*g_1_*−*g_3_*
**1^+^** (exp.)	2.054^[a]^	2.041, 2.041, 2.128	0.087
**1^+^** (DFT calc.)	2.062	2.018, 2.052, 2.165	0.147
**2^+^** (exp.)	2.058	1.984, 2.096, 2.164	0.180
**2^+^** (DFT calc.)	2.085	1.992, 2125, 2.140	0.148

[a] Hyperfine splitting: 4.96 mT (1×^195^Pt, I=1/2, 33.8 % nat. abundance).

**Figure 9:**
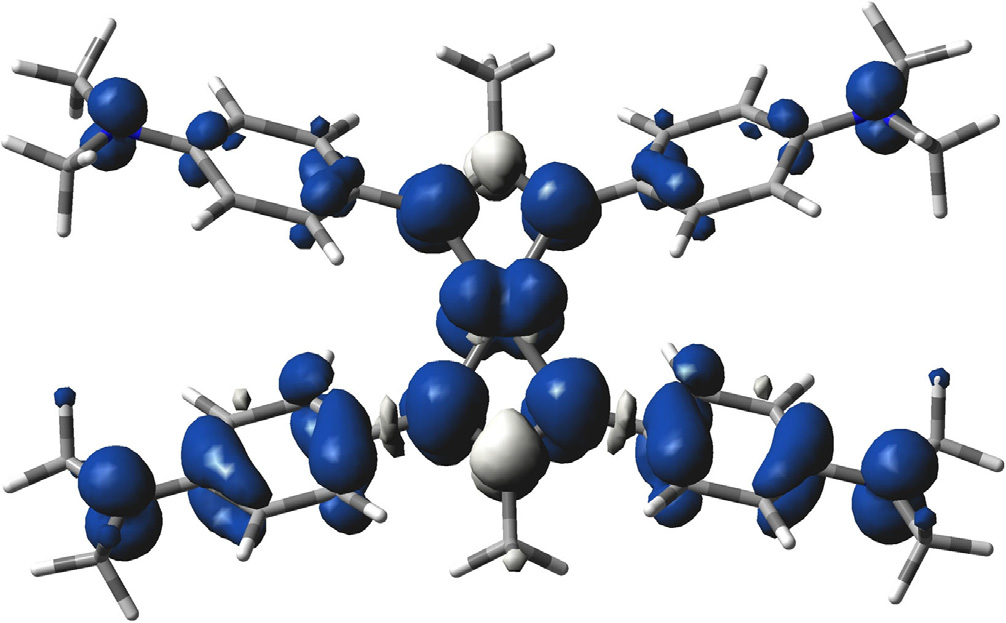
DFT (ADF) calculated spin densities for [PtL_2_]^+^=**1^+^**.

Both **1^+^** and **2^+^** display EPR signals at room temperature in CH_2_Cl_2_ solution. While **2^+^** did not exhibit any hyperfine information for the signal at *g=*2.058 (Figure 8 a), the electrogenerated complex **1^+^** (*g=*2.054) showed a ^195^Pt isotope coupling of 4.96 mT (Figure 6)—a value which compares well with that of other Pt^II^ complexes with anion radical ligands[^16^, ^17^] and signifies mostly but not exclusively ligand‐based spin. In frozen solution **1^+^** exhibits a relatively small *g* anisotropy *g_1_*‐*g_3_*=0.087 (Figure 7). In agreement, the DFT calculation yielded spin densities of 0.12 for platinum and 0.44 for each ligand (Figure 9). Replacing Pt by Pd in the geometry‐optimized spin density calculation yields a very similar structure for the hypothetical [PdL_2_]^+^ with slightly less spin density (0.096) on the metal (Figure S2). Figures S3 and S5 depict the frontier orbitals of **1** and **2**, Figures S4 and S6 show corresponding orbitals for the cations **1^+^** and **2^+^**. During oxidation of **1**=[PtL_2_] an electron is withdrawn from the HOMO, localized primarily on the L^−^ ligands, with contributions from the Pt d_π_ orbital. Calculated *g_1_*, *g_2_*, *g_3_* values of 2.018, 2.052, 2.165 and a *g_iso_* of 2.062 reasonably reproduce the experimental *g* values of 2.041, 2.041, 2.128 and *g_iso_*=2.054 (Table S4).

Obviously, the [Pt^II^(L^0.5−^)_2_]^+^ formulation with L delocalized spin is more appropriate than an alternative [Pt^III^(L^−^)_2_]^+^. Mononuclear platinum(III) species are rare[^18^, ^19^] and should display extremely wide g anisotropy due to the high spin‐orbit coupling constant of that heavy metal ion.[^18^, ^19^, ^20^] The second oxidation separated by 0.36 V from the first is then attributed to formation of a diradical complex [(L )Pt^II^(L )]^2+^ which, however, proved to be too labile for further study.

**Figure 10:**
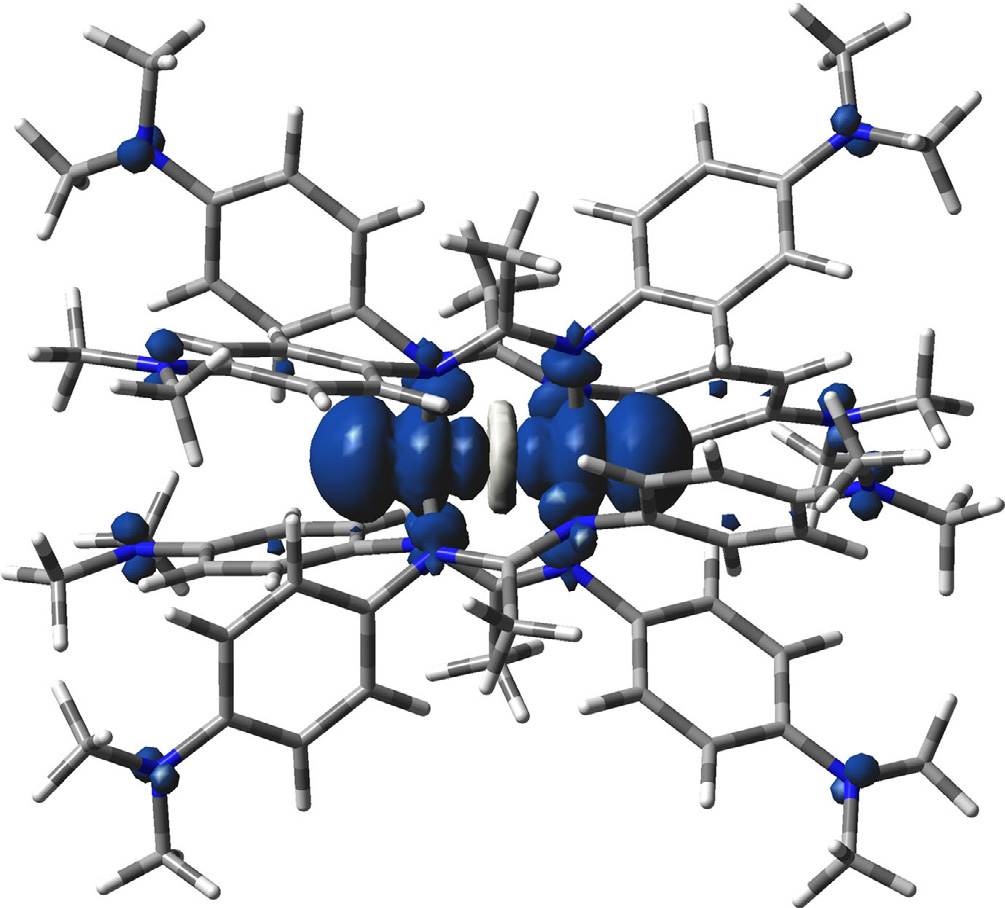
DFT (ADF) calculated spin densities for [Pd_2_L_4_]^+^=**2^+^**.

In the case of **2** an electron is withdrawn from the Pd dσ* HOMO, producing spin densities for **2^+^** of 0.34 at each Pd center (Figure 10). Calculated *g_1_*, *g_2_*, *g_3_* and *g_iso_* values of 1.992, 2.125, 2.140 and 2.085 are comparable with the experimental *g* parameters of 1.984, 2.096, 2.164 and 2.058 (=*g_iso_)*, respectively (Table 1).

The EPR data of **2^+^** in frozen solution (Figure 8 b, Table 1) thus suggest significant amounts of spin on the mixed‐valent dimetal unit (Pd_2_^5+^).^[15]^ However, the smaller spin‐orbit coupling constant of Pd vs. Pt^[20]^ leads to a less pronounced *g* anisotropy despite metal‐based spin.

The cationic form is thus assigned an oxidation state formulation [(Pd^2.5^)_2_(L^−^)_4_]^+^, in agreement with earlier assignments for Pd_2_^5+^ species.^[15]^ This result proves that the dipalladium(II) unit *Pd_2_* ^*4*+^ is giving up an electron easier than the oxidizable^[8]^ ligands L^−^. These appear to be oxidized only in further steps which include more than one electron according to the cyclic voltammetry experiment (Figure 5).

**Figure 11:**
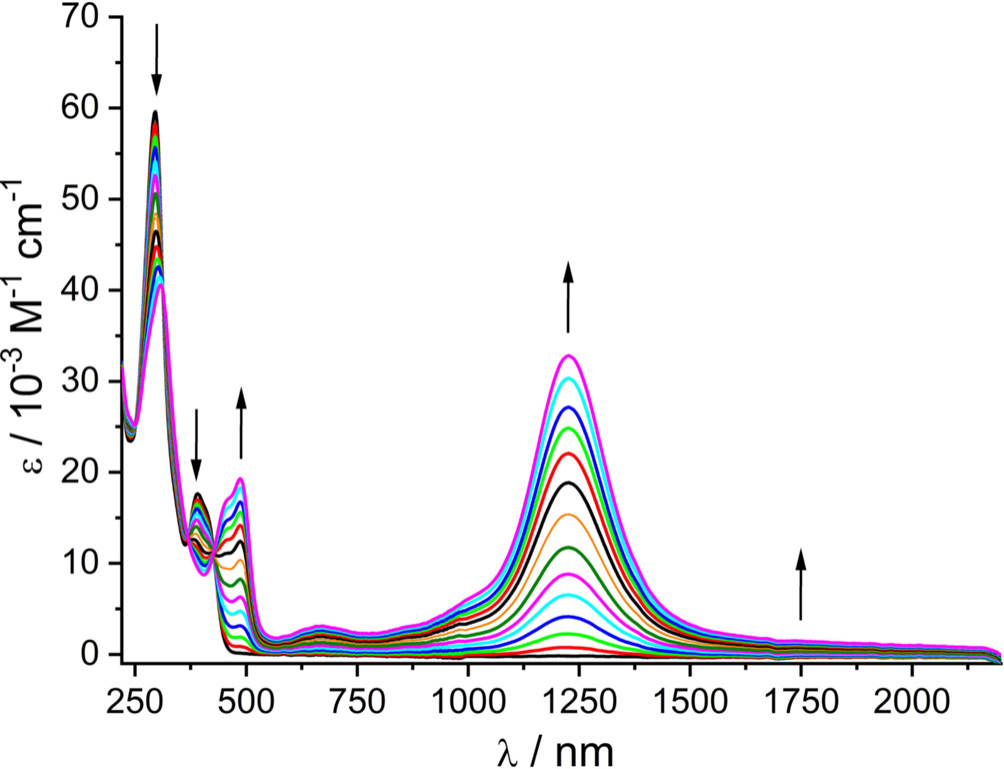
UV‐vis‐NIR spectroelectrochemical response on oxidation of [PtL_2_] in CH_2_Cl_2_/0.1 m Bu_4_NPF_6_.

**Figure 12:**
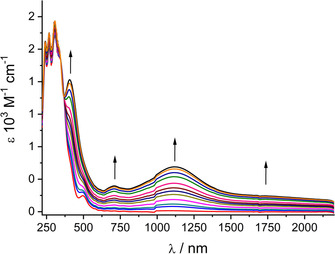
UV‐vis‐NIR spectroelectrochemical response on oxidation of [Pd_2_L_4_] in CH_2_Cl_2_/0.1 m Bu_4_NPF_6_.

Using an OTTLE cell^[16]^ it was possible to study UV‐vis‐NIR absorption the response of reversible one‐electron oxidation for both **1** and **2** (Figures 11 and 12, Tables S5 and S6).

Table 2 lists the TD DFT calculated lowest lying transitions for [PtL_2_]=**1** and its oxidized product **1^+^**. The two lowest allowed transitions of the neutral species are formed by excitations from the HOMO with partial Pt d_π_ contribution into the purely l‐based LUMO or LUMO+1. The intense NIR transition calculated for the oxidized complex at 1206 nm can be characterized as an IL/LMCT transition from the l‐localized βHOMO‐1 into a partly metal localized βLUMO.

**Table 2:** TD‐DFT (PBE0/PCM‐CH_2_Cl_2_) calculated lowest lying transitions of [PtL_2_]^*n*+^=**1^+^** with significant oscillator strengths^[a]^.

*n*	State^b^	Mainly contributing excitations [%]	Calc. transition energy^[c]^ [eV] (nm)	Calc. osc. str.	Exp. abs. maximum [eV] (nm)^[c]^	Molar extinction [m ^−1^ cm^−1^]
0	b^1^A	67 (HOMO→LUMO)	2.83 (439)	0.150	n. o.	
	c^1^A	75 (HOMO→LUMO+1)	3.34 (371)	0.351	3.17 (391)	17 800
1	b^2^A	99 (βHOMO→βLUMO)	0.50 (2495)	0.109	0.73 (1700)	sh
	c^2^A	99 (βHOMO‐1→βLUMO)	1.03 (1206)	0.474	1.01 (1225)	33 000
	d^2^A	45 (αHOMO→αLUMO)	2.87 (432)	0.090	2.56 (485)	19 500

[a] MOs involved in calculated transitions are depicted in Figures S3 and S4. [b] Lower case letter indicates order, upper index multiplicity, and symbol A symmetry of given state. [c] Wavelengths in parentheses.

**Figure 13:**
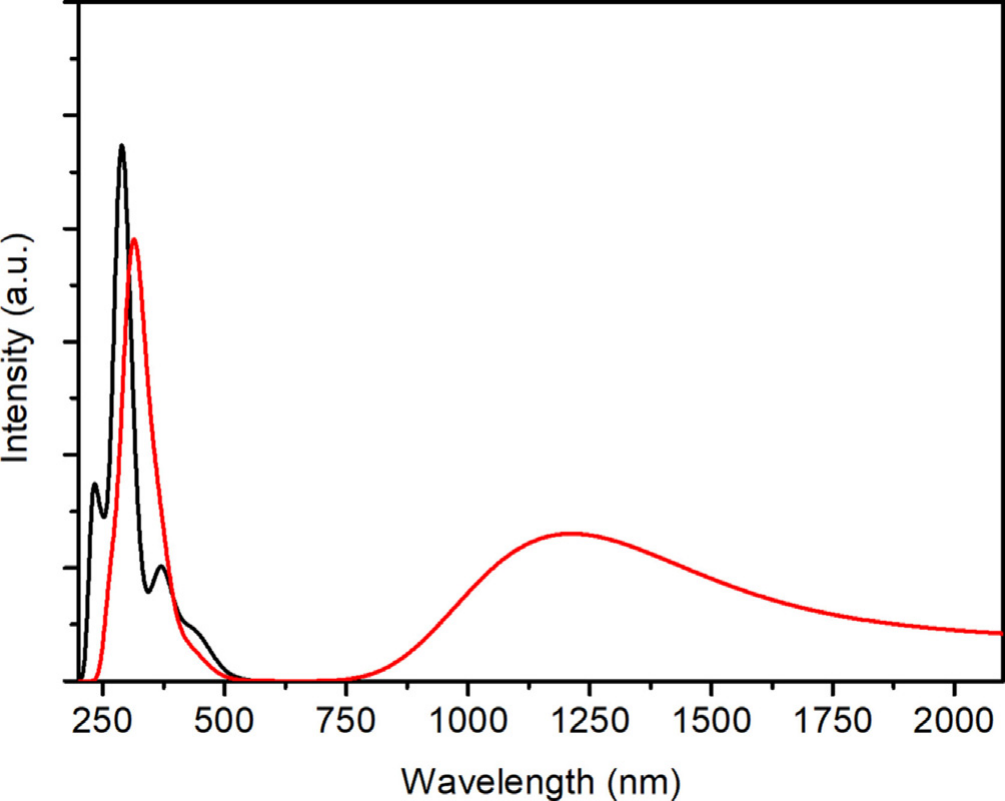
TD DFT simulated UV‐vis‐NIR spectra of **1** (black line) and **1^+^** (red line).

**Figure 14:**
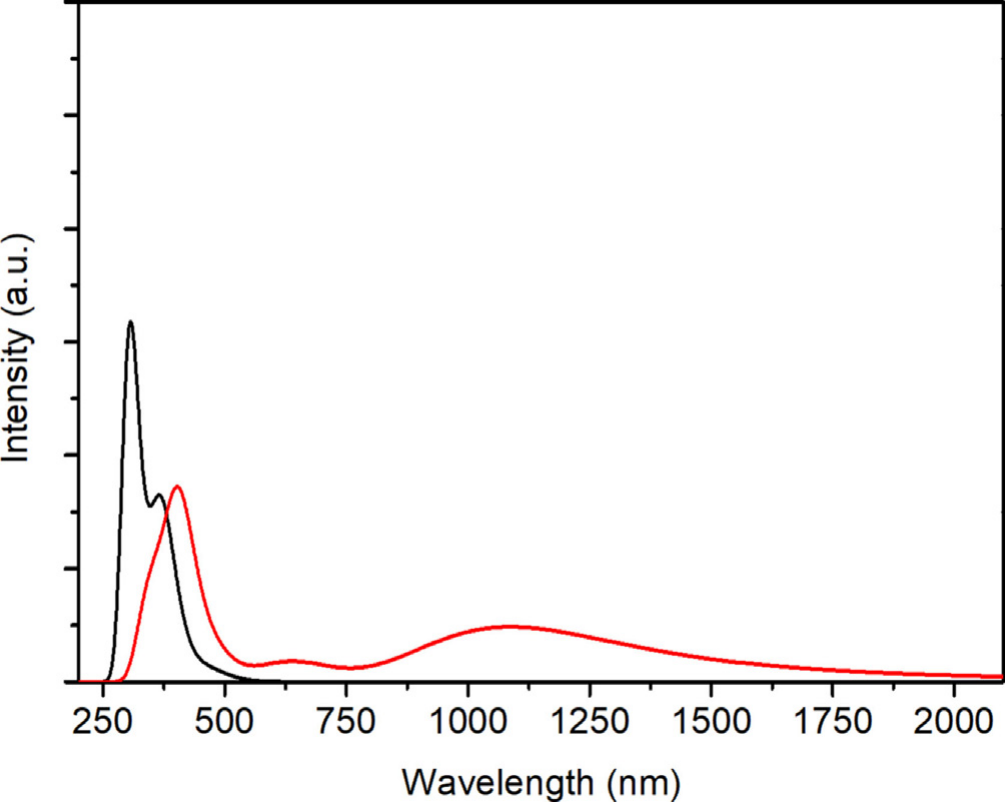
TD DFT simulated UV‐vis‐NIR spectra of **2** (black line) and **2^+^** (red line).

Table 3 summarizes the calculated lowest lying transitions for **2** (*n*=0,1). The lowest allowed transitions of **2** correspond to excitations from the Pd d_σ_* HOMO into the LUMO or LUMO+1. The transition calculated for the oxidized species **2^+^** at 1608 nm is an excitation from the ligand localized βHOMO into the Pd d_σ_* βLUMO. The calculated feature around 1100 nm is formed by several IL excitations from the occupied l‐based β spin‐orbitals into the βLUMO. Simulated spectra of both complexes and their oxidized products depicted in Figures 13 and 14 reproduce the experimental spectra, including the lower intensity in the NIR region of **2** in comparison with **1**.

**Table 3:** TD‐DFT (PBE0/PCM‐CH_2_Cl_2_) calculated Lowest Lying Transitions of [Pd_2_L_4_]^*n*+^=**2** ^***n*****+**^ with significant oscillator strengths^[a]^.

*n*	State^[b]^	Mainly contributing excitations [%]	Calc. transition energy^[c]^ [eV] (nm)	Calc. osc. str.	Exp. abs. maximum [eV] (nm)^[c]^	Molar extinction [m ^−1^ cm^−1^]
0	b^1^A	79 (HOMO‐1→LUMO)	2.56 (484)	0.035	2.50 (497)	2600
	e^1^A	48 (HOMO‐2→LUMO+1)	3.35 (370)	0.192	3.64 (340)	sh
	f^1^A	47 (HOMO‐3→LUMO+1)	3.38 (367)	0.230	4.05 (306)	27 100
1	b^2^A	96 (βHOMO→βLUMO)	0.77 (1608)	0.013	0.72 (1730)	sh
	c^2^A	97 (βHOMO‐3→βLUMO)	1.11 (1119)	0.059	1.11 (1120)	7100
	d^2^A	98 (βHOMO‐4→βLUMO)	1.13 (1102)	0.046		
	e^2^A	89 (βHOMO‐5→βLUMO)	1.16 (1068)	0.058		
	g^2^A	91 (βHOMO‐9→βLUMO)	1.91 (650)	0.057	1.76 (705)	4000
	k^2^A	35 (αHOMO −4→αLUMO) 31 (βHOMO‐3→βLUMO+1)	3.02 (410)	0.169		
	l^2^A	52 (αHOMO −5→αLUMO) 29 (βHOMO −4→βLUMO+1)	3.04 (408)	0.208	3.04 (408)	20 000

[a] MOs involved in calculated transitions are depicted in Figures S5 and S6. [b] Lower case letter indicates order, upper index multiplicity, and symbol A symmetry of given state. [c] Wavelengths in parentheses.

## Conclusions

Remarkably different products were obtained when reacting the easily oxidizable HL/L^−1^ system with dichlorometal compounds of platinum or palladium. The mononuclear Pt^II^ complex **1**=[PtL_2_] tolerates two κ^2^ *N*,*N’*‐L^−^ ligands with very small bite angles of 62.9(2)° in severely constrained four‐membered chelate rings. In contrast, a paddle‐wheel situation **2**=[Pd_2_L_4_] with more relaxed five‐membered ‐Pd‐N‐C‐N‐Pd‐ rings, bridging[^9^, ^12^, ^21^] amidinato ligands and significant metal–metal distances were found with the lighter homologue, comparable to other Pd_2_^4+^ species.[^13^, ^14^, ^15^] Although palladium and platinum have very similar radii,^[13]^ the higher number of available orbitals in the heavier homologue seems to favor greater structural flexibility, here the tolerance of unusually small angles.

In addition to the unexpected constitutional and structural differences of the two M^II^ compounds **1** and **2**, the oxidation behavior showed distinct alternatives. In agreement with the established^[8]^ facile oxidation of the ligand L^−^ to the radical L^.^ the compound **1** exhibits stepwise ligand‐based electron removal, avoiding an oxidation of the metal to the uncommon Pt^III^ state or formation of a diplatinum compound.^[22]^ Conversely, the Pd_2_^4+^ complex **2** undergoes one‐electron oxidation mainly at the dimetal unit to yield a symmetrically mixed‐valent Pd_2_^5+^ species.^[15]^

Summarizing, the results described in this article illustrate a dipalladium paddle‐wheel structure with metal–metal based electron transfer activity while the heavier metal platinum avoids the metal–metal interaction in favor of a classical but strained chelate arrangement, the one‐electron oxidation involving the noninnocent ligands L^−^ with their propensity to form neutral radical ligands L .

## Experimental Section

### Physical measurements

EPR spectra in the X band were recorded with a Bruker EMX System. ^1^H NMR spectra were taken on a Bruker AC 250 spectrometer. IR spectra were obtained using a Nicolet 6700 FT‐IR instrument; solid state IR measurements were performed with an ATR unit (smart orbit with diamond crystal). UV/Vis‐NIR absorption spectra were recorded on J&M TIDAS and Shimadzu UV 3101 PC spectrophotometers. Cyclic voltammetry was carried out in 0.1 m Bu_4_NPF_6_ solutions using a three‐electrode configuration (glassy carbon working electrode, Pt counter electrode, Ag/AgCl reference) and a PAR 273 potentiostat and function generator. The ferrocene/ferrocenium (Fc/Fc^+^) couple served as internal reference. Spectroelectrochemistry was performed using an optically transparent thin‐layer electrode (OTTLE) cell.^[23]^ A two‐electrode capillary served to generate intermediates for X band EPR studies.^[24]^

### X‐ray crystallographic studies

Crystallization of **1** and **2** was achieved by slow evaporation of CH_2_Cl_2_/n‐hexane solutions. Single crystals of [(**2**)][B{3,5‐(CF_3_)_2_C_6_H_3_}_4_] were obtained by slow diffusion of *n*‐hexane into a CH_2_Cl_2_ solution. X‐ray diffraction data were collected using a Bruker Kappa Apex2duo diffractometer at 100 K. The structures were solved and refined by full‐matrix least‐squares techniques on *F* ^*2*^ using the SHELX‐97 program.[^25^, ^26^] The absorption corrections were done by the multi‐scan technique. All data were corrected for Lorentz and polarization effects, and the non‐hydrogen atoms were refined anisotropically. Hydrogen atoms were included in the refinement process as per the riding model.

Deposition numbers 2005227, 2005235 and 2005236 contain the supplementary crystallographic data for this paper. These data are provided free of charge by the joint Cambridge Crystallographic Data Centre and Fachinformationszentrum Karlsruhe Access Structures service.

### Synthesis of [PtL_2_] (1) and [Pd_2_L_4_] (2)

Mixtures of *cis*‐[Pt(DMSO)_2_Cl_2_]^[27]^ or *cis*‐[Pd(MeCN)_2_Cl_2_] (Aldrich) (0.24 mmol), HL (140 mg, 0.48 mmol)^[8]^ and Cs_2_CO_3_ (782 mg, 2.40 mmol) were stirred in acetone at 60 °C for 15 h. The solutions were filtered through celite and the solvent was removed. The remaining solids were purified by column chromatography (CH_2_Cl_2_/MeOH 9:1) to afford the products. **[PtL_2_]**: Yield 150 mg (80 %). Anal. calcd for C_36_H_48_N_8_Pt: C 54.88, H 6.14, N 14.22. Found: C 54.47, H 5.83, N 13.92. ESI‐MS *m*/*z* calcd (found): 786.36 (786.36). ^1^H NMR (250 MHz, CD_2_Cl_2_): *δ*=1.79 (s, 6 H), 2.82 (s, 24 H), 6.42 (m, 8 H), 6.62 ppm (m, 8 H). **[Pd_2_L_4_]**: Yield 140 mg (84 %). Anal. calcd for C_144_H_192_N_16_Pd_2_: C 61.84, H 6.92, N 15.44. Found: C 62.07, H 6.77, N 15.44. ESI‐MS *m*/*z* calcd (found): 1394.58 (1394.58). ^1^H NMR (250 MHz, CD_2_Cl_2_): *δ*=1.57 (s, 12 H), 2.91 (s, 48 H), 6.19 (m, 16 H), 6.52 ppm (m, 16 H).

### Single crystals of [Pd_2_L_4_]{B[3,5‐(CF_3_)_2_C_6_H_3_]_4_}

A mixture of [Pd_2_L_4_] (50 mg, 0.036 mmol) and [Fe(C_5_H_5_)_2_]{B[3,5‐(CF_3_)_2_C_6_H_3_]_4_}^[28]^ (38 mg, 0.036 mmol) was stirred in 10 mL CH_2_Cl_2_ for 1 h at room temperature. The resulting solution was layered with 40 mL of *n*‐hexane and the resulting solid recrystallized from CH_2_Cl_2_/*n*‐hexane (Yield: 62 mg, 77 %). Single crystals were obtained by slow diffusion of *n*‐hexane into a CH_2_Cl_2_ solution. For EPR data see Table 1 in main text.

### Quantum chemical calculations

The electronic structures of [PtL_2_] and [Pd_2_L_4_] and of their oxidized forms were calculated by density functional theory (DFT) methods using the Gaussian 09^[29]^ program package. Calculations employed the Perdew, Burke, Ernzerhof ^[30, 31]^ PBE0 hybrid functional (G09/PBE0). Geometry optimization was followed by vibrational analysis in order to characterize stationary states. For H, C and N atoms polarized triple‐ ζ basis sets 6–311G(d),^[32]^ together with quasirelativistic effective core pseudopotentials and corresponding optimized sets of basis functions for Pt and Pd were used.[^33^, ^34^] The solvent was described by the polarizable calculation model (PCM).^[35]^ Electronic spectra were calculated by the TD‐DFT method at geometries optimized with PCM correction.

EPR parameters were calculated with the ADF 2016.06 program package.^[36]^ Within ADF Slater type orbital (STO) basis sets of triple‐ζ quality with two polarization functions for Pt and Pd atoms and of triple‐ζ quality with one polarization function for the remaining atoms were employed, together with the PBE0 hybrid functional. The scalar relativistic (SR) zero order regular approximation (ZORA) was used within ADF calculations. The g tensor was obtained from a spin‐nonpolarized wave function after incorporating the spin‐orbit (SO) coupling using the first‐order perturbation theory from a ZORA Hamiltonian in the presence of a time‐independent magnetic field.^[37]^

## Conflict of interest

The authors declare no conflict of interest.

## Supporting information

As a service to our authors and readers, this journal provides supporting information supplied by the authors. Such materials are peer reviewed and may be re‐organized for online delivery, but are not copy‐edited or typeset. Technical support issues arising from supporting information (other than missing files) should be addressed to the authors.

Supplementary
