# Supplementary material for: Structural and Oxidation State Alternatives in Platinum and Palladium Complexes of a Redox‐Active Amidinato Ligand
Source: Chemistry. 2021 Jan 12;27(10):3374–81. doi: 10.1002/chem.202003636 (PMC7986709; doi:10.1002/chem.202003636)
Supplement: Supplementary file 1 — Supplementary [file CHEM-27-3374-s001.pdf]

Chemistry–A European Journal

Supporting Information

Structural and Oxidation State Alternatives in Platinum and Palladium Complexes of a Redox-Active Amidinato Ligand

Fabian Ehret,[a] Vasileios Filippou,[a] Svenja Blickle,[a] Martina Bubrin,[a] Stanislav Záliš,[b] and Wolfgang Kaim*[a]

Table S1. Crystallographic data for **1**×CH_2Cl_2, **2** and [**2**][B{3,5-$(CF_3)_2C_6H_3$}$_4$].

	**1**	**2**	[**2**][B{3,5-$(CF_3)_2C_6H_3$}$_4$]
empirical formula	$C_{38}H_{50}Cl_2N_8Pt$	$C_{72}H_{89}N_{16}Pd_2$	$C_{110}H_{114}BF_{24}N_{16}Pd_2$
formula weight	884.85	1391.39	2339.78
wavelength (Å)	0.71073	1.54178	0.71073
crystal system	monoclinic	monoclinic	monoclinic
space group	*C*2/*m*	*P*2/*n*	*C*2/*c*
a (Å)	24.345(5)	17.6921(15)	30.942(6)
b (Å)	7.5156(17)	19.5211(17)	21.858(4)
c (Å)	11.526(2)	20.7805(17)	21.539(4)
β (°)	113.104(9)	91.570(5)	133.17(3)
volume ($Å^3$)	1939.7(7)	7174.3(11)	10624(3)
Z	2	4	4
density (calcd) (Mg/m^3)	1.515	1.288	1.463
absorption coeff. (mm^{-1})	3.792	4.444	0.437
F(000)	892	2900	4796
crystal size (mm^3)	0.24 × 0.17 × 0.08	0.23 × 0.07 × 0.05	0.36 × 0.22 × 0.16
theta range for data collection (°)	1.82 to 28.43	2.26 to 66.37	1.30 to 28.35
index ranges	$-32 \le h \le 32$, $-10 \le k \le 9$, $-14 \le l \le 15$	$-17 \le h \le 20$, $-22 \le k \le 22$, $-17 \le l \le 24$	$-40 \le h \le 41$, $-29 \le k \le 29$, -28<=*l*<=28
reflections collected	9189	101396	106365
independent reflections	2580 [R(int) = 0.0573]	12261 [R(int) = 0.0642]	13264 [R(int) = 0.0852]
completeness to theta = 25.00° (%)	99.4		100.0
completeness to theta = 66.37° (%)		97.2	
absorption correction	numerical	semi-empirical from equivalents	semi-empirical from equivalents
max. and min. transmission	0.8004 and 0.5087	0.7528 and 0.5295	0.7397 and 0.6959
data / restraints / parameters	2580 / 6 / 191	12261 / 0 / 833	13264 / 0 / 703
goodness-of-fit on F^2	1.039	1.057	1.010

final R indices [I>2σ (I)]	R1 = 0.0432, wR2 = 0.1085	R1 = 0.0357, wR2 = 0.0886	R1 = 0.0416, wR2 = 0.0871
R indices (all data)	R1 = 0.0453, wR2 = 0.1104	R1 = 0.0472, wR2 = 0.0937	R1 = 0.0863, wR2 = 0.1067
largest diff. peak and hole (e•Å^{-3})	3.224 and -1.658	0.662 and -0.668	0.868 and -0.659

Table S2. The comparison of selected experimental and DFT calculated bond lengths (Å) and angles (°) for $[PtL_2]$ = **1**.

bond lengths	exp.	calc.
Pt1-N2	2.049(5)	2.050
Pt1-N2#1	2.049(5)	2.050
Pt1-N1#1	2.056(6)	2.050
Pt1-N1	2.056(6)	2.050
Pt1-C1#1	2.527(7)	2.535
Pt1-C1	2.527(7)	2.535
N2-C1#1	1.320(9)	1.329
N2-C10	1.414(9)	1.394
N1-C1	1.324(9)	1.329
N1-C3	1.387(8)	1.394
N3-C6	1.395(8)	1.389
C3-C4	1.351(10)	1.396
C4-C5	1.402(13)	1.386
C6-C5	1.400(11)	1.407
C3-C4A	1.436(10)	1.397
C4A-C5A	1.381(13)	1.384
C6-C5A	1.399(10)	1.406
bond angles		
N2-Pt1-N1#1	62.9(2)	63.0
N2-Pt1-N1	117.1(2)	117.0
N1#1-Pt1-N1	180.0(5)	180.0
N1-C1-N2	108.2(6)	107.3

Table S3. The comparison of selected experimental and DFT calculated bond lengths (Å) and angles (°) for $[Pd_2L_4]^{n+}$ = $\mathbf{2^{n+}}$ (n=0, 1).

	n=0		n=1	
bond lengths	exp.	calc.	exp.	calc.
Pd1-Pd1#1	2.5432(4)	2.567	2.4384(9)	2.454
Pd2-Pd2#2	2.5648(2)			
Pd1-N1	2.049(5)	2.051	2.033(2)	2.040
Pd2-N15	2.043(2)			
Pd1-N2	2.047(2)	2.052	2.035(2)	2.042
Pd2-N11	2.047(2)			
Pd1-N5	2.053(2)	2.052	2.028(2)	2.041
Pd2-N10	2.050(2)			
Pd1-N6	2.049(2)	2.052	2.042(2)	2.043
Pd2-N14	2.055(2)			
N1-C1	1.342(4)	1.333	1.332(4)	1.329
N1-C3	1.410(4)	1.406	1.423(4)	1.414
N2-C1#1	1.325(4)	1.329	1.332(4)	1.327
C3-C4	1.384(4)	1.400	1.391(4)	1.398
C3-C9	1.402(4)	1.400	1.391(4)	1.401
C5-C4	1.402(4)	1.387	1.379(4)	1.388
C5-C6	1.391(5)	1.409	1.405(4)	1.411
C6-C7	1.391(5)	1.407	1.408(4)	1.412
C9-C7	1.378(4)	1.390	1.381(4)	1.386
N3-C6	1.395(8)	1.393	1.390(4)	1.376
bond angles				
N2-Pd1-N6	171.83(9)	171.8	173.84(10)	174.5
N11-Pd2-N10	171.40(10)			

N2-Pd1-N1	91.19(10)	89.8	89.91(9)	177.9
N15-Pd2-N10	91.06(9)			
N6-Pd1-N1	88.31(10)	89.6	90.22(9)	80.9
N15-Pd2-N11	88.57(9)			
N2-Pd1-N5	88.55(10)	89.8	89.91(9)	99.1
N10-Pd2-N14	88.62(9)			
N6-Pd1-N5	90.64(10)	89.8	89.27(9)	99.1
N11-Pd2-N14	90.40(9)			
N1-Pd1-N5	170.81(9)	170.7	173.61(10)	173.2
N15-Pd2-N14	171.02(10)			
N2-Pd1-Pd1#1	86.35(6)	85.9	86.86(7)	87.3
N14-Pd2-Pd2#2	85.57(7)			
N6-Pd1-Pd1#1	85.48(6)	85.4	86.86(7)	86.6
N15-Pd2-Pd2#2	85.46(7)			
N1-Pd1-Pd1#1	84.96(6)	85.9	87.16(8)	87.3
N11-Pd2-Pd2#2	85.45(7)			
N5-Pd1-Pd1#1	85.86(6)	85.9	86.75(7)	86.6
N10-Pd2-Pd2#2	85.95(7)			

Table S4. The comparison of selected experimental and DFT calculated bond lengths (Å) and angles (°) for $[Pd_2L_4]^{n+}$, n = 0,1.

	n = 0		n=0		n=1	
bond lengths, "Pd2"		bond lengths, "Pd1"	exp.	calc.	exp.	calc.
Pd(2)-Pd(2)#2	2.5648(4)	Pd(1)-Pd(1)#1	2.5432(4)	2.567	2.4384(9)	2.454
Pd(2)-N(15)	2.043(2)	Pd(1)-N(1)	2.049(5)	2.051	2.033(2)	2.040
Pd(2)-N(11)	2.047(2)	Pd(1)-N(2)	2.047(2)	2.052	2.035(2)	2.042
Pd(2)-N(10)	2.050(2)	Pd(1)-N(5)	2.053(2)	2.052	2.028(2)	2.041
Pd(2)-N(14)	2.055(2)	Pd(1)-N(6)	2.049(2)	2.052	2.042(2)	2.043
N(14)-C(61)	1.329(3)	N(1)-C(1)	1.342(4)	1.333	1.332(4)	1.329
N(11)-C(50)	1.419(4)	N(1)-C(3)	1.410(4)	1.406	1.423(4)	1.414
N(10)-C(40)#2	1.336(4)	N(2)-C(1)#1	1.325(4)	1.329	1.332(4)	1.327
C(78)-C(73)	1.386(4)	C(3)-C(4)	1.384(4)	1.400	1.391(4)	1.398
C(73)-C(74)	1.384(5)	C(3)-C(9)	1.402(4)	1.400	1.391(4)	1.401
C(78)-C(77)	1.385(5)	C(5)-C(4)	1.402(4)	1.387	1.379(4)	1.388
C(77)-C(76)	1.385(5)	C(5)-C(6)	1.391(5)	1.409	1.405(4)	1.411
C(75)-C(76)	1.397(5)	C(6)-C(7)	1.391(5)	1.407	1.408(4)	1.412
C(74)-C(75)	1.393(5)	C(9)-C(7)	1.378(4)	1.390	1.381(4)	1.386
N(17)-C(76)	1.407(4)	N(3)-C(6)	1.395(8)	1.393	1.390(4)	1.376
		bond angles				
N(11)-Pd(2)-N(10)	171.40(10)	N(2)-Pd(1)-N(6)	171.83(9)	171.8	173.84(10)	174.5
N(15)-Pd(2)-N(10)	91.06(9)	N(2)-Pd(1)-N(1)	91.19(10)	89.8	89.91(9)	177.9
N(11)-Pd(2)-N(14)	90.40(9)	N(6)-Pd(1)-N(1)	88.31(10)	89.6	90.22(9)	80.9
N(15)-Pd(2)-N(11)	88.57(9)	N(2)-Pd(1)-N(5)	88.55(10)	89.8	89.91(9)	99.1
N(10)-Pd(2)-N(14)	88.62(9)	N(6)-Pd(1)-N(5)	90.64(10)	89.8	89.27(9)	99.1
N(15)-Pd(2)-N(14)	171.02(10)	N(1)-Pd(1)-N(5)	170.81(9)	170.7	173.61(10)	173.2

N(15)-Pd(2)-Pd(2)#2	85.46(7)	N(2)-Pd(1)-Pd(1)#1	86.35(6)	85.9	86.86(7)	87.3
N(11)-Pd(2)-Pd(2)#2	85.45(7)	N(6)-Pd(1)-Pd(1)#1	85.48(6)	85.4	86.86(7)	86.6
N(10)-Pd(2)-Pd(2)#2	85.95(7)	N(1)-Pd(1)-Pd(1)#1	84.96(6)	85.9	87.16(8)	87.3
N(14)-Pd(2)-Pd(2)#2	85.57(7)	N(5)-Pd(1)-Pd(1)#1	85.86(6)	85.9	86.75(7)	86.6

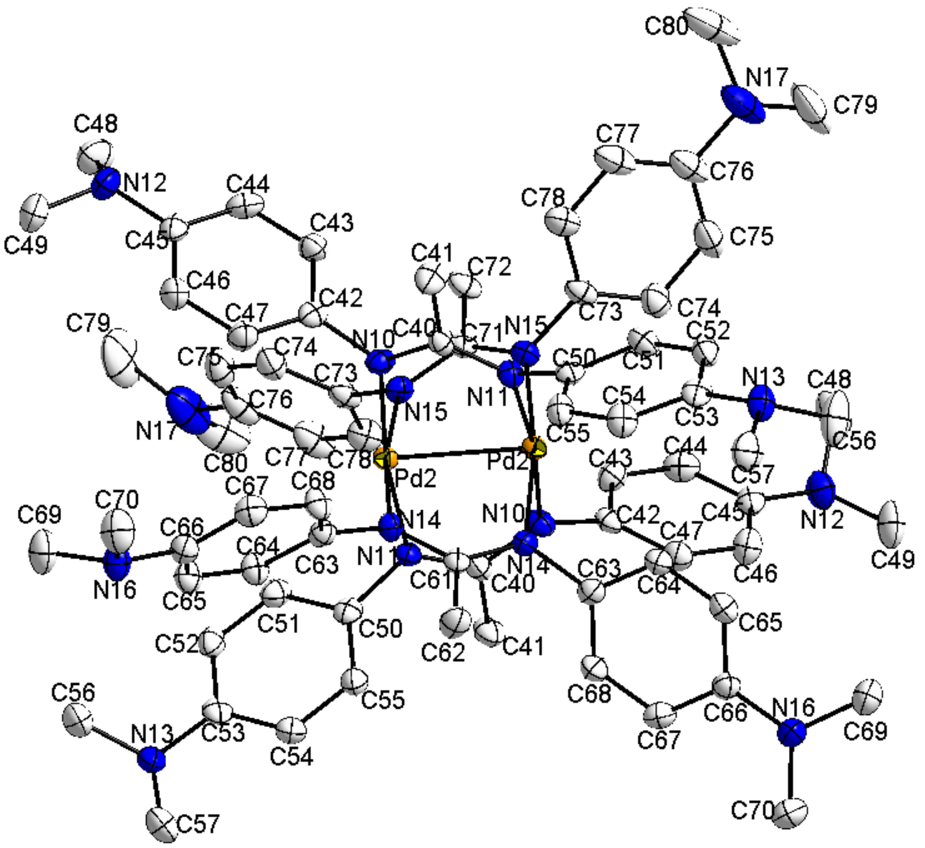

Figure S1. Structure of molecule "Pd2" in the crystal of **2**.

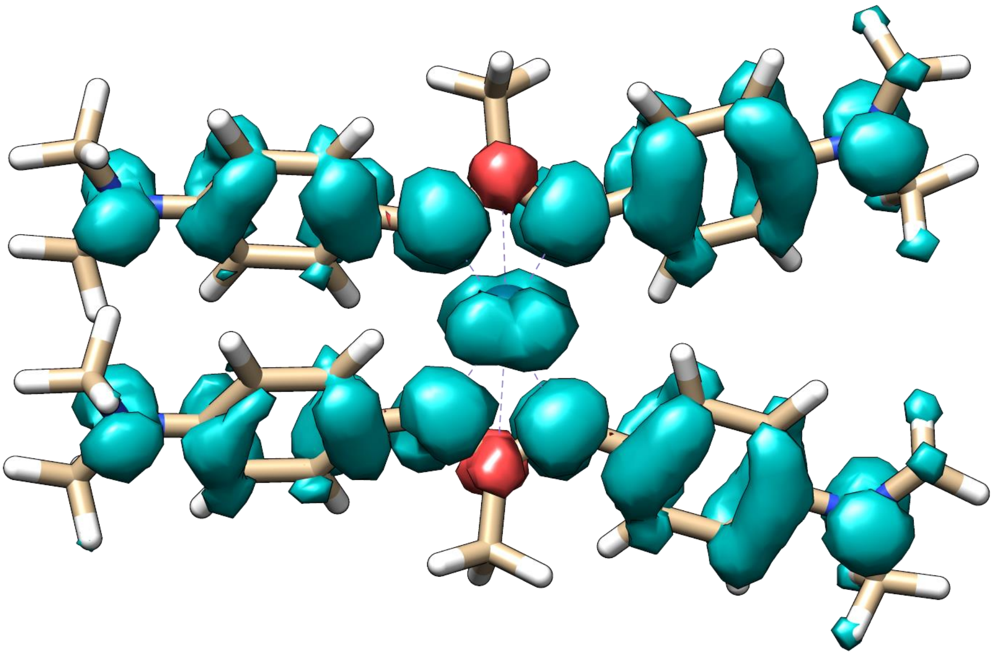

Figure S2. DFT-Calculated spin density for $[PdL_2]^+$ (Pd: 9.6%).

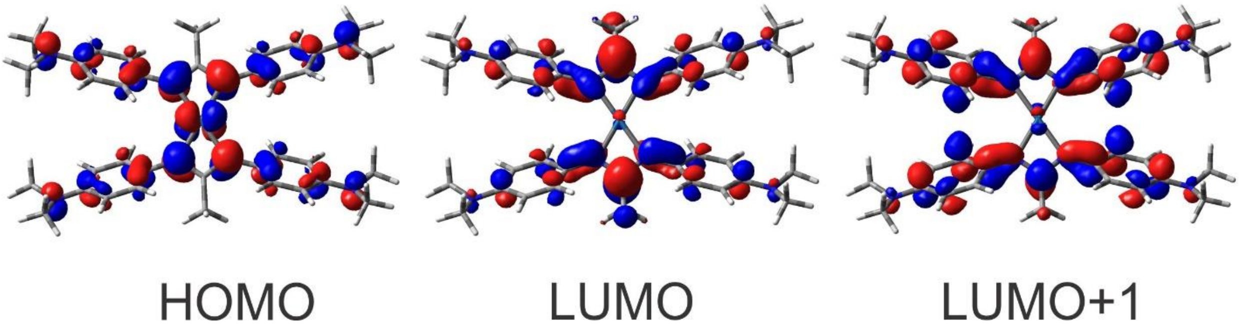

Figure S3. The representation of FMOs of $[PtL_2]$.

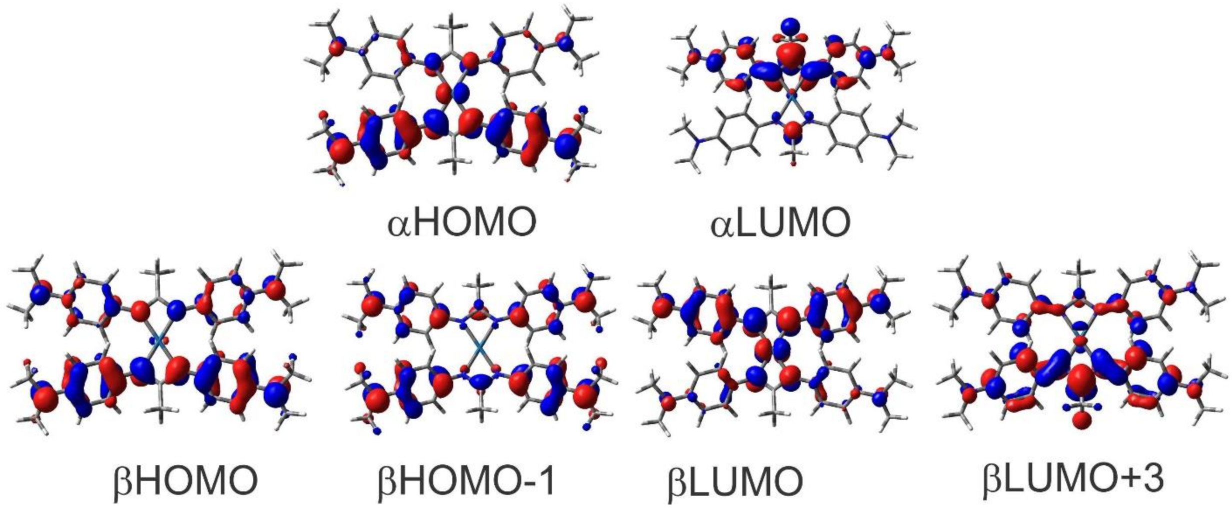

Figure S4. The representation of FMOs of $[PtL_2]^+$.

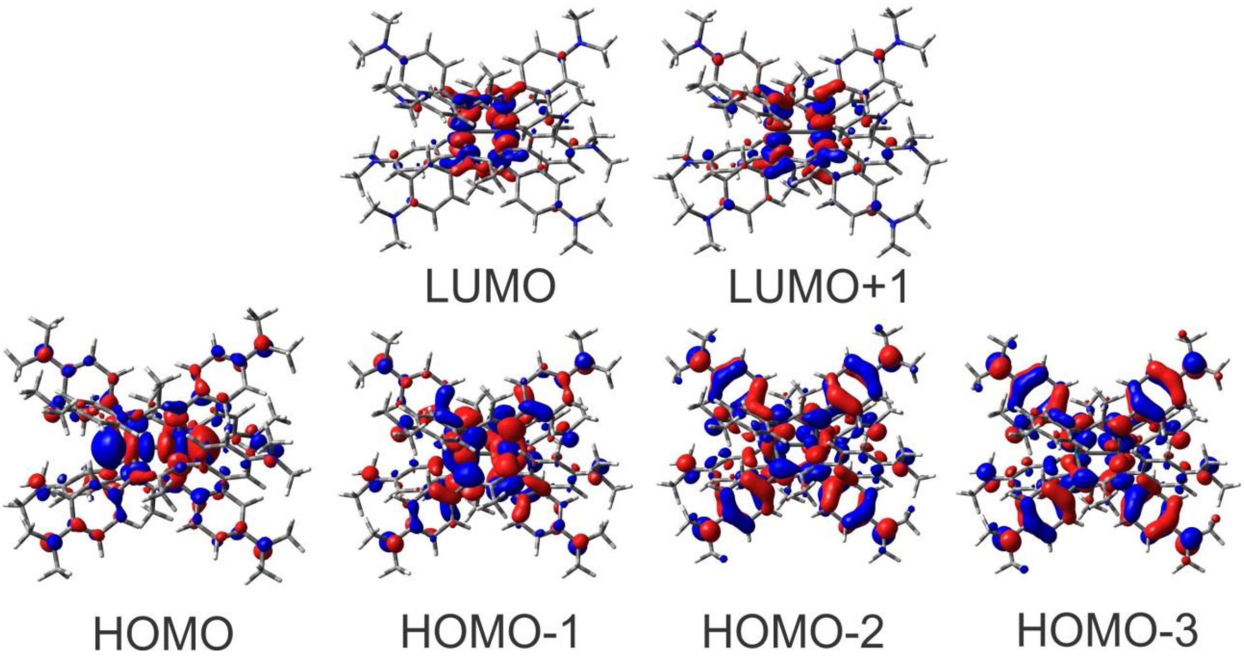

Figure S5. The representation of FMOs of $[Pd_2L_4]$.

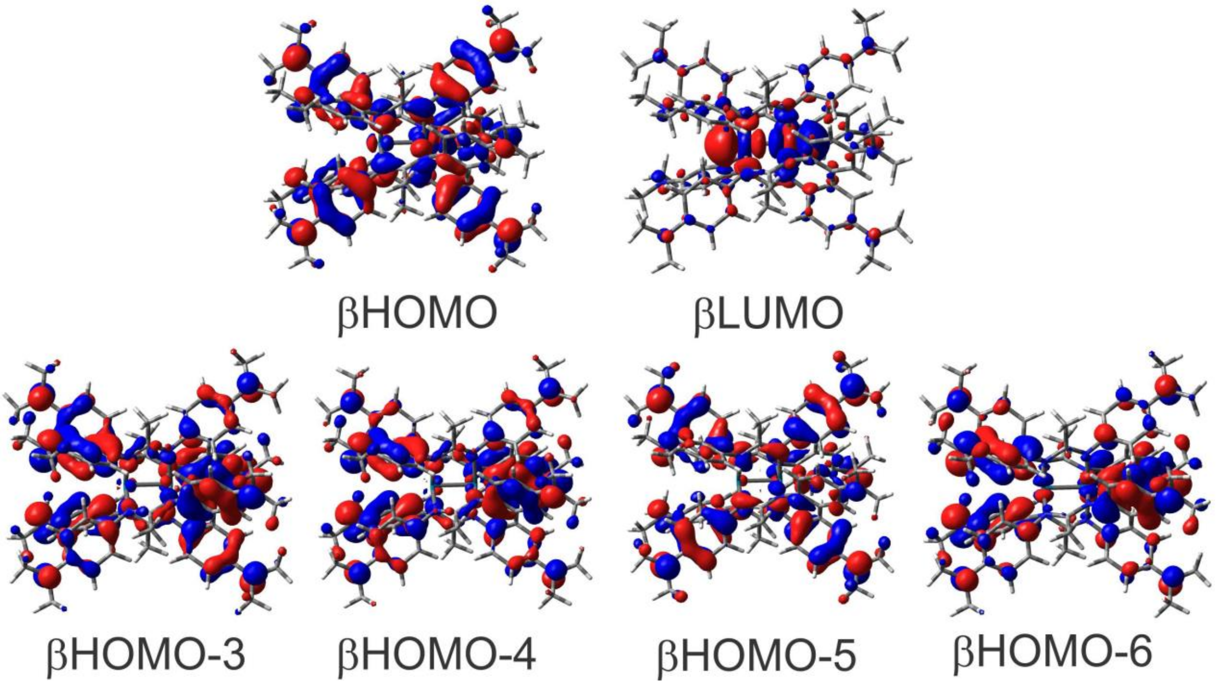

Figure S6. The representation of FMOs of $[Pd_2L_4]^+$.
